# Wireless Reconfigurable RF Detector Array for Focal and Multiregional Signal Enhancement

**DOI:** 10.1109/access.2020.3011905

**Published:** 2020-07-24

**Authors:** WEI QIAN, XIN YU, CHUNQI QIAN

**Affiliations:** 1 Department of Electrical and Computer Engineering, Michigan State University, East Lansing, MI 48824, USA; 2 Martinos Center for Biomedical Imaging, Massachusetts General Hospital, Charlestown, MA 02129, USA; 3 Department of Radiology, Michigan State University, East Lansing, MI 48824, USA

**Keywords:** Sensor arrays, nonlinear circuits, inductive power transmission, magnetic resonance

## Abstract

Wirelessly Amplified NMR Detectors (WAND) can utilize wireless pumping power to amplify MRI signals *in situ* for sensitivity enhancement of deep-lying tissues that are difficult to access by conventional surface coils. To reconfigure between selective and simultaneous activation in a multielement array, each WAND has a dipole resonance mode for MR signal acquisition and two butterfly modes that support counter-rotating current circulation. Because detectors in the same row share the same lower butterfly frequency but different higher butterfly frequency, a pumping signal at the sum frequency of the dipole mode and the higher butterfly mode can selectively activate individual resonators, leading to 4-fold sensitivity gain over passive coupling. Meanwhile, a pumping signal at the sum frequency of the dipole mode and the lower butterfly mode can simultaneously activate multiple resonators in the same row, leading to 3-fold sensitivity gain over passive coupling. When multiple rows of detectors are parallelly aligned, each row has a unique lower butterfly frequency for consecutive activation during the acquisition interval of the others. This wireless detector array can be embedded beneath a headpost that is normally required for multi-modal brain imaging, enabling easy reconfiguration between focal imaging of individual vessels and multiregional mapping of brain connectivity.

## I. INTRODUCTION

To decipher brain dynamics from micro to mesoscales, functional MRI (fMRI) has been performed on rodent brain in combination with optic-mediated brain stimulation and recording [1]–[3]. Normally, such a multi-modal imaging platform consists of a head-post that is fixed on top of the skull to restrict head motion during MR experiments and to secure fiber-optics insertion into the brain cortex through a tiny orifice. Because the headpost is normally concealed to prevent infection, a conventional surface coil needs to be placed above the headpost, leading to limited detection sensitivity as a result of its large distance separation from the brain cortex. To improve MR detection sensitivity of deep-lying tissues, inductively coupled detectors [4]–[14] can be placed near the region of interest to relay locally detected MR signals to the external surface coil. However, passive inductive coupling may have low efficiency especially when the internal detector has a large distance separation from the external coil. To overcome signal transmission attenuation over large distance separations, Wirelessly Amplified NMR Detectors (WAND) [15]–[18] have been developed that can be embedded beneath the headpost for proximal detection of the brain cortex. Without the need for wired connections or internal batteries, the WAND can utilize wireless pumping power to amplify locally detected MR signals *in situ,* before wirelessly transmitting them to an externally separated receiver coil that is connected to the MRI console.

Most previous versions of WAND were designed for single-element operation, making them suitable to highlight a specific sample region that is comparable to their own dimensions. To observe a larger field-of-view (FOV), it is in principle possible to increase the detector’s dimension, but at the expense of reduced focal sensitivity. To enlarge the FOV without sacrificing detection sensitivity of localized regions, multiple detectors were concatenated [19], each of which was activatable by a unique pumping frequency. Although concatenated resonators could be individually activated to highlight their respective regions, simultaneous activation of multiple resonators required concurrent application of multiple pumping signals (an energy-inefficient procedure that was tedious to implement). Therefore, it would be ideal if only one pumping signal is required to wirelessly reconfigure the entire detector array between selective and simultaneous activation.

In this work, we are going to demonstrate the design concept of such a wireless reconfigurable detector array. In this array, each element is activatable by two pumping frequencies, one is unique to each resonator while the other is common to multiple detectors in the same row. As a result, each detector can be selectively activated at its unique pumping frequency, and multiple detectors can be simultaneously activated at their common pumping frequency. Unlike conventional detector arrays where individual detectors are cable-connected to dedicated receiver channels on the MRI console, this wireless reconfigurable detector array can selectively or simultaneously relay amplified signals into a single external receiver, thus providing a convenient method to multiplex receiver channels without the need for expensive multichannel upgrade. This wireless reconfigurable detector array will be useful for both high-resolution imaging of single-vessels in focal brain cortex and multiregional mapping of the whole-brain functional connectivity, paving way for future utilization for sensitivity-enhanced multi-modal imaging.

## II. OPERATION PRINCIPLE

**FIGURE 1.**
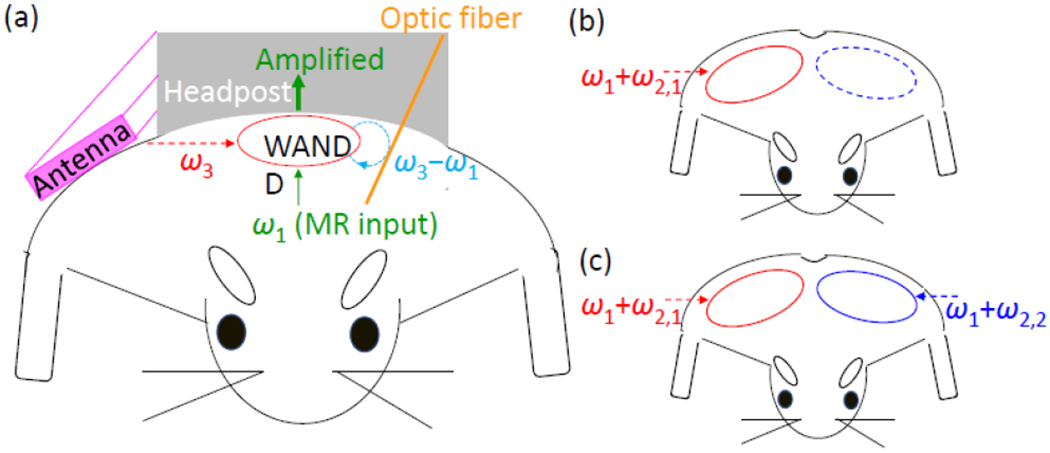
(a) A headpost (gray) is fixed on top of a rodent skull to secure optical fiber (orange) insertion into the brain. To improve detection sensitivity of the brain cortex, a Wireless Amplified NMR Detector is embedded beneath the headpost to observe the cortex region from immediate adjacency. In the presence of a pumping signal provided by the antenna (pink), the WAND can utilize wireless pumping power to amplify MR signals through multi-stage signal mixing. (b) In a detector array, by making each element sensitive to a unique pumping frequency, only one detector is activated at a time to highlight a specific brain region. (c) To activate multiple detectors together, the previous approach was to apply multiple pumping signals simultaneously.

A WAND can be implemented as a nonlinear multi-frequency resonator [20], [21]. By making the sum of its two lower resonance frequencies approximately equal to the highest resonance frequency, the resonator can utilize the wireless pumping signal at *ω*_3_ (that is provided near its highest resonance frequency) to amplify the MR signal at *ω*_1_ (that is detected near one of its lower resonance frequencies). Although the signal created at the difference frequency *ω*_3_ − *ω*_1_ is not directly detected, this “idler” signal can mix back with the pumping signal at *ω*_3_ to create an amplified signal at *ω*_1_ that is much larger than the original signal (Fig. 1A). Such multi-stage signal mixing process can occur only if the circuit has an extra resonance mode near *ω*_3_ − *ω*_1_ to support efficient current circulation of “idler” signal. Most previous versions of wireless amplifiers were simplified to degenerate resonators where the MR and idler signals shared the same resonance mode [15]–[18]. These simplified designs, however, required the pumping frequency at *ω*_3_ to be approximately twice the MR frequency at 2*ω*_1_, thus precluding the frequency-specific activation of individual detectors when multiple detectors were concatenated in an array. To overcome this limitation, a more recent approach was to use multiple non-degenerate resonators [19], each of which can support idler signal circulation at a unique frequency *ω*_2,*i*_. As a result, each resonator could be individually activated by a specific pumping frequency at *ω*_1_ + *ω*_2,*i*_ (Fig. 1b). However, to activate multiple detectors together, simultaneous application of multiple pumping signals was required (Fig. 1c), making iterative adjustment of individual pumping signals tedious to implement.

**FIGURE 2.**
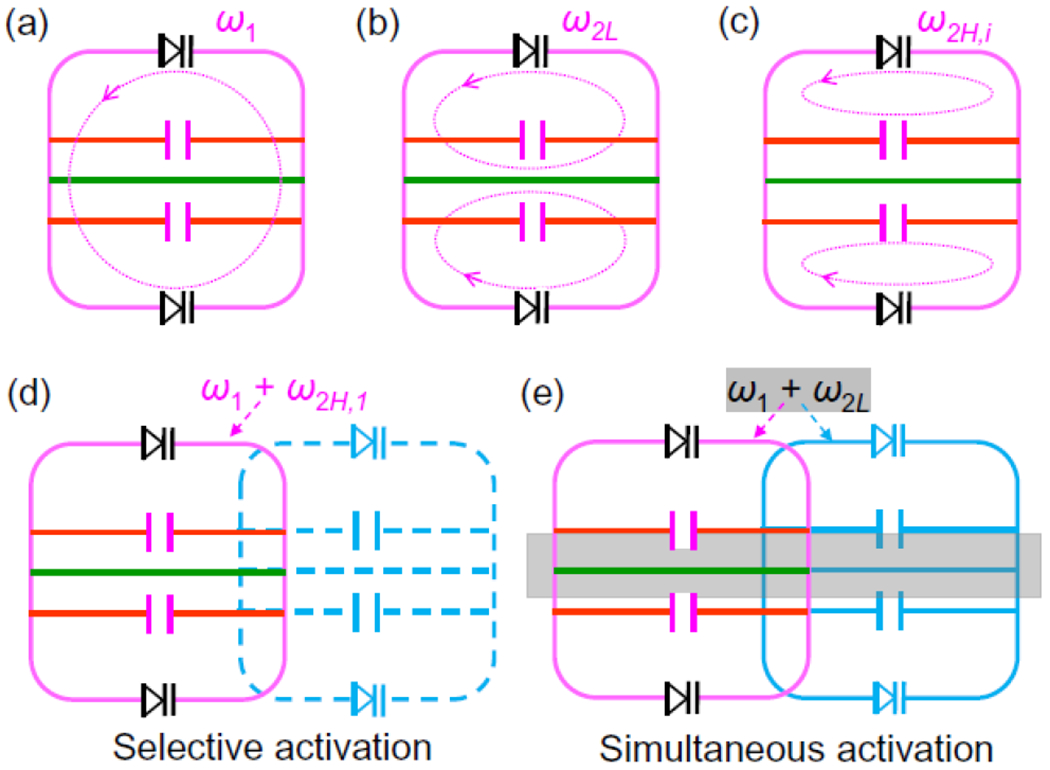
To make a parametric resonator activatable by two pumping frequencies, it needs to have a dipole mode (a), a lower butterfly mode (b) and a higher butterfly mode (c). As a result, a pumping signal at the sum frequency of the dipole and higher butterfly mode would selectively activate an individual detector (d). Alternatively, a pumping signal at the sum frequency of the dipole and lower butterfly mode would simultaneously activate multiple detectors (e).

In this work, we will incorporate an additional “idler” resonance mode into each detector to make multiple resonators activatable by the same pumping frequency, thus greatly simplifying the operation procedure. As shown in Fig. 2, a single parametric resonator can be activated by two pumping frequencies. This resonator consists of a square conductor (shown in pink) that is symmetrically bridged by two variable capacitors (shown in black) connected in head-to-head configuration, leading to a dipole mode resonance that can support current flow around its peripheral edges (Fig. 2a). This mode is normally utilized to detect MR signals at the Larmor frequency *ω*_1_, just like a conventional surface coil. To support “idler” current flow, a “lower butterfly” resonance mode at *ω*_2*L*_ is created by bridging the virtual voltage grounds of the dipole mode with a continuous conductor in the center, enabling counter-rotating current flow through this center conductor (green line in Fig. 2b). As a result, MR signals can be amplified by the energy converted from the pumping signal at *ω*_1_+*ω*_2*L*_. For a square resonator split by a continuous conductor in its center, *ω*_2*L*_ ≈ 3*ω*_1_/4 (a relation that will be explained in Eq. A10 of the appendix.). To make the resonator activatable at a different pumping frequency, another “higher butterfly” mode at *ω*_2*H*_ is created by two conductors (red lines in Fig. 2c) that are symmetrically placed above and below the center conductor, each with a chip capacitor in its center gap to enable counter-rotating current flow within the topmost and bottom circuit meshes. Similar as the lower butterfly mode, this higher butterfly mode (Fig. 2c) also has anti-symmetric current flows. But unlike the lower butterfly mode that has most of its current circulating through the continuous center conductor (Fig. 2b), the higher butterfly mode has most of its current circulating through the gapped conductors split by chip capacitors. As a result, the resonance frequency of this higher butterfly mode is approximately proportional to the inverse square root of this chip capacitance (Eq. A11 in the Appendix). When multiple resonators of different higher butterfly frequencies are partially overlapped for inductive decoupling [22], each element can be selectively activated by a pumping signal at *ω_i_* + *ω*_2*H,i*_ (Fig. 2d), where *ω*_2*H,i*_ is the resonator’s higher butterfly frequency. Alternatively, multiple resonators can be simultaneously activated by a pumping signal at *ω*_1_ + *ω*_2*L*_ (Fig. 2e), where ω_2*L*_ is the “lower butterfly” frequency for all resonators.

**FIGURE 3.**
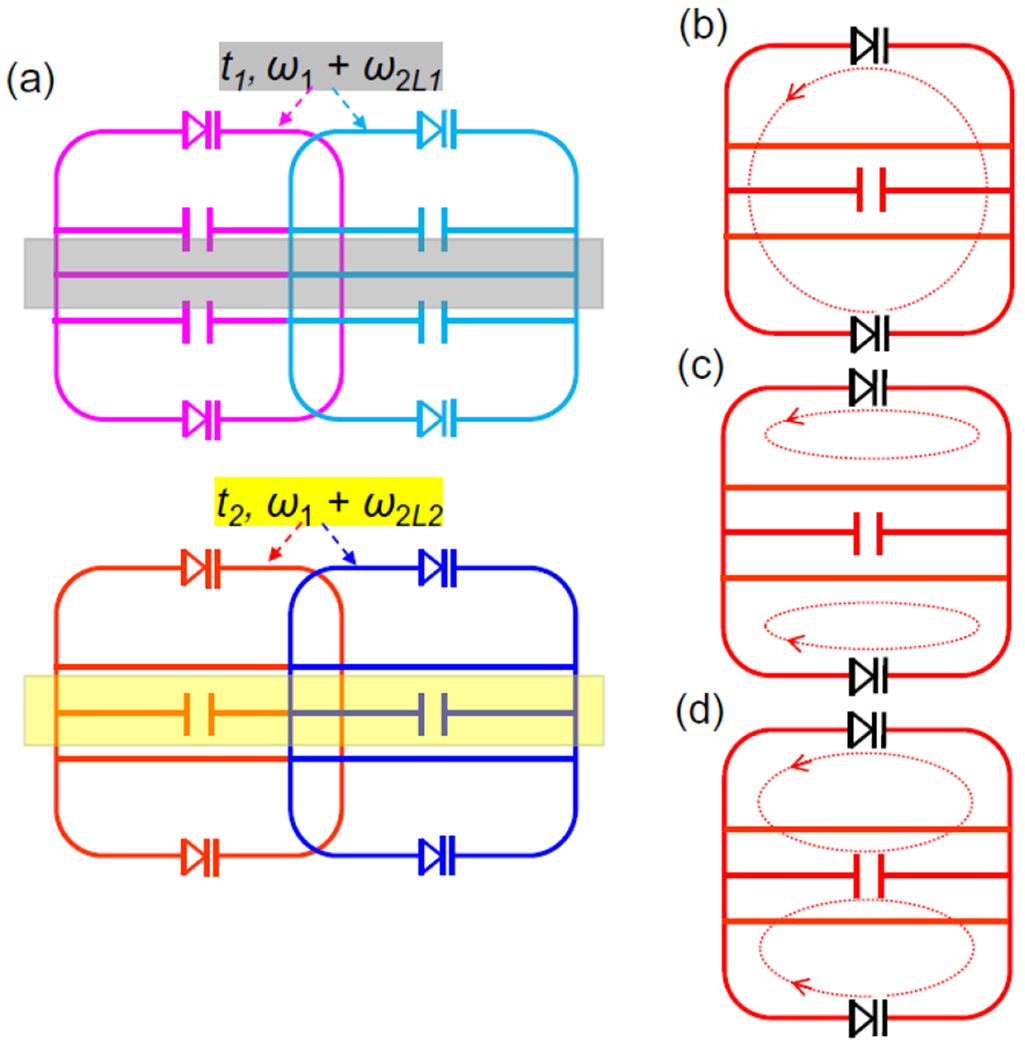
(a) Two rows of parametric resonators that are parallelly aligned to be consecutively activatable by their respective pumping frequencies. (b) Compared to resonators in the first row, resonators in the second row have a dipole mode that retains similar current pattern. (c) But their lower butterfly mode has circulation current restricted to the upper and lower meshes. (d) Their higher butterfly mode has circulation current going through the center conductor.

To create a two-dimensional wireless detector array, we will adjacently place a second row of parametric resonators in parallel with the first row (Fig. 3a), where each row can be consecutively activated during the acquisition interval of the other. To obtain a different “lower butterfly” frequency for this second row, each detector is slightly modified (Fig. 3b) to have its center conductor split by a chip capacitor and a pair of continuous conductors symmetrically displaced from the center. As a result, the dipole mode current pattern is mostly unaffected, but the lower butterfly mode (Fig. 3c) has most of its circulation current restricted to the topmost and the bottom most circuit meshes, leading to larger resonance frequency compared to resonators in the first row. As a general guideline, when the height of each half circuit is scaled down by a factor of u, the lower butterfly frequency will be scaled up by a factor of $1∕\sqrt[{4}]{u}$. This scaling relation will be explained in Eq. A12 of the appendix. On the other hand, the higher butterfly mode (Fig. 3d) has most of its circulation current passing through the center conductor, where the splitting capacitor can be conveniently replaced to adjust the higher butterfly resonance without affecting the other two modes. This higher butterfly frequency will again be proportional to the inverse square root of splitting capacitor, as will be shown in Eq. A13 in the Appendix.

## III. MATERIALS AND METHODS

### A. INDIVIDUAL WIRELESS AMPLIFIER

**FIGURE 4.**
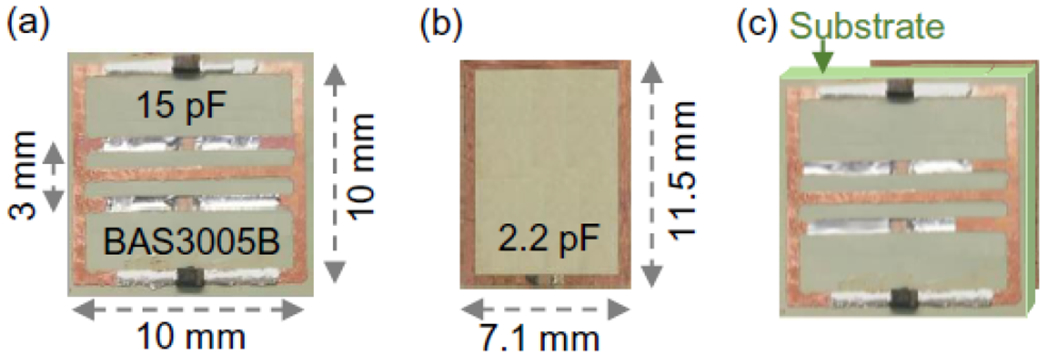
Diagrams of (a) the parametric resonator (b) the passive coupler and (c) two resonators overlapped across a substrate.

The parametric resonator was etched on copper-clad polyimide as a 10 × 10-mm^2^ rectangular conductor loop with a continuous conductor in its center (Fig. 4a). The widths of conductor strips were 0.6 mm. The conductor gaps on the bottom most and topmost legs were filled by Schottky diodes (BAS3005B, Infineon, Germany) as variable capacitors, while the conductor gaps adjacently below and above the center conductor were filled by 15-pF chip capacitors. As a result, the parametric resonator had a lower-frequency butterfly mode at 226.0 MHz, a higher-frequency butterfly mode at 498.3 MHz and a dipole mode resonance at 300.9 MHz that was slightly above the Larmor frequency at 7T (300.3 MHz). To improve the circuit’s coupling efficiency with the pumping signal, the parametric resonator was overlaid on top of a passive resonator with a dimension of 11.5 × 7.1-mm^2^ whose conductor gap was filled by a 2.2-pF chip capacitor. The thickness of the sandwiching substrate between these two resonators was empirically adjusted to about 0.6 mm until the dipole mode resonance of the parametric resonator was adjusted to 300.3 MHz. Meanwhile, the passive coupler had a resonance peak at 799 MHz, which was close enough to the optimal pumping frequency estimated as the sum of the Larmor frequency (300.3 MHz) and the higher butterfly frequency (498.3 MHz). As a result, the resonator could be efficiently activated by less than 10 mW of power applied on an antenna that was 3-cm separated from the resonator’s circuit edge.

### B. FIRST ROW OF AMPLIFIER ARRAY

**FIGURE 5.**
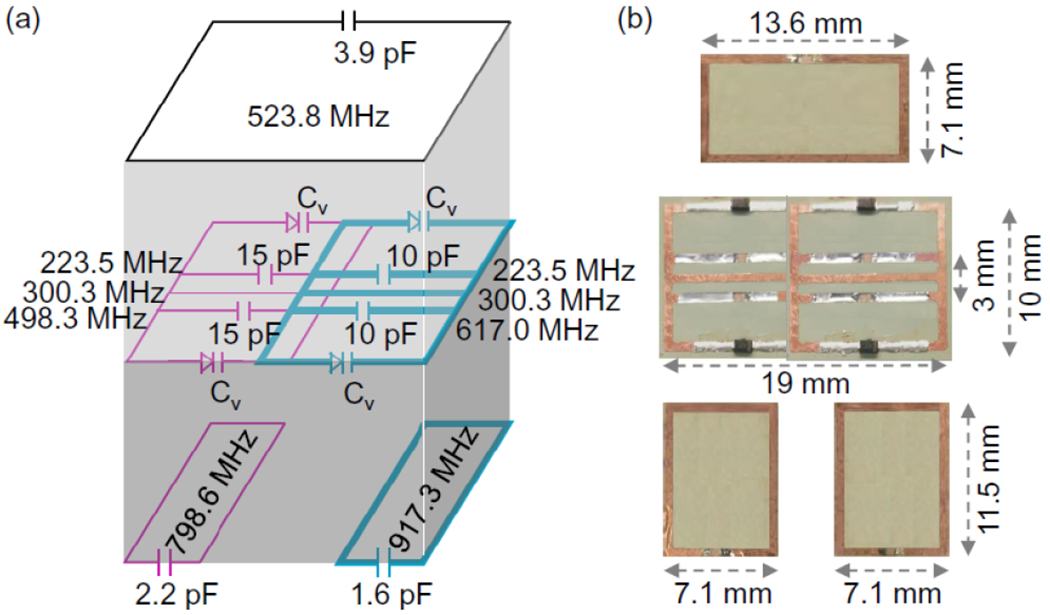
(a) Diagrams of parametric resonators that were overlaid with passive couplers. (b) Pictures of the partially overlapped parametric resonators (in the middle) that were overlaid on top of passive couplers (at the bottom) to improve pumping power efficiency for individual activation. Another single-loop coupler was optionally overlaid on top of both parametric resonators to improve power efficiency for simultaneous activation.

In another concatenated resonator (cyan in Fig. 5a), the same circuit pattern was used. But the gapped conductors immediately beneath and above the center conductor were replaced by two 10-pF chip capacitors, leading to a higher butterfly frequency at 617.0 MHz. Moreover, to improve the circuit’s coupling efficiency with the pumping signal, the parametric resonator was overlaid on top of a passive resonator with a dimension of 11.5 × 7.1-mm^2^ whose conductor gap was filled by a 1.6-pF chip capacitor. The substrate thickness of the sandwiching substrate was empirically adjusted to about 0.6 mm when the dipole mode resonance frequencies were 300.3 MHz for the parametric resonator and 917 MHz for the passive coupler.

When these two almost identical resonators were concatenated (Fig. 5), they were inductively decoupled by experimentally adjusting their partial overlap area until they showed up an unsplit resonance peak around 300.3 MHz. Meanwhile, the lower-frequency butterfly mode was down shifted to 223.5 MHz due to residual coupling between the butterfly modes of these two resonators. (The higher-frequency butterfly modes of both resonators were virtually unaffected due to their large frequency separation.) Therefore, simultaneous activation of these two resonators required optimal pumping frequency at 523.8 MHz (as the sum of 300.3 MHz and 223.5 MHz). To improve the coupling efficiency at 523.8 MHz for both parametric resonators, another passive resonator was optionally introduced with a dimension of 13.6 × 7.1 mm^2^. The gap of its conductor loop was filled with a 3.9-pF chip capacitor to obtain a resonance frequency at MHz, which was close enough to the optimal pumping frequency at 523.8 MHz. By placing the passive coupler on top of both parametric resonators via a 2-mm thick poly-imide substrate, the resonance frequencies of the coupled assembly were virtually unaffected, due the relatively large substrate thickness. Meanwhile the required pumping power at 523.8 MHz decreased from about 50 mW to about 10 mW when the antenna was placed at a 3-cm distance separation from the edge of the coupled resonators.

### C. SECOND ROW OF AMPLIFIER ARRAY

**FIGURE 6.**
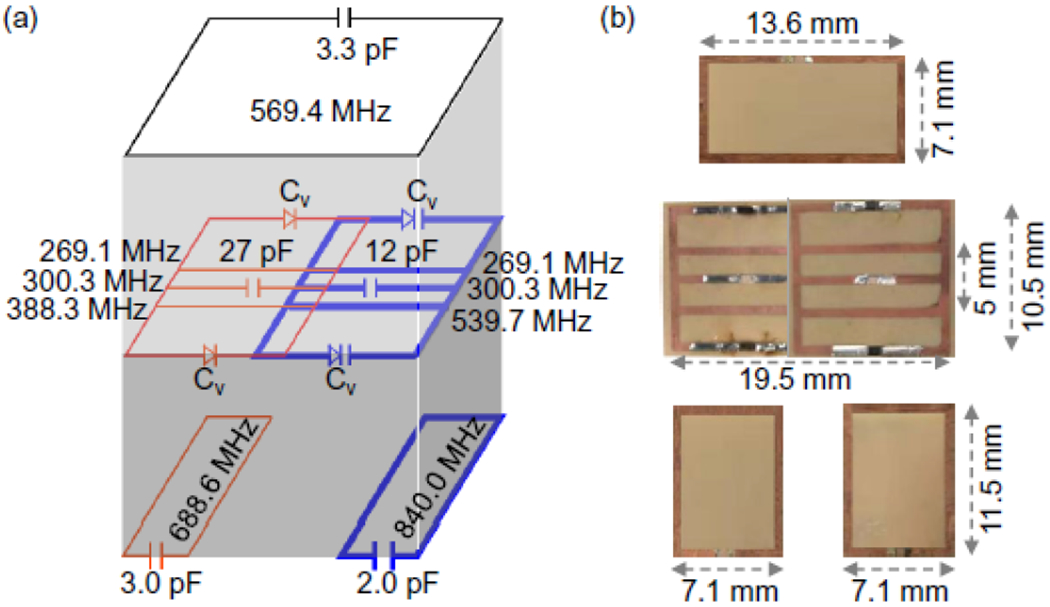
(a) Diagrams of slightly modified parametric resonators with increased “lower butterfly” resonance frequency. (b) Pictures of the coupled pair of parametric resonators (in the middle) that were overlaid on top of passive couplers (at the bottom) to improve pumping power efficiency for individual activation. Another passive coupler was optionally overlaid on top of both parametric resonators to improve power efficiency for simultaneous activation.

Fig. 6 showed the second row of parametric resonators that could be simultaneously activated at a different pumping frequency. Just like the first row mentioned above, this second row included two partially overlapped resonators, while each resonator was etched as a 10.5 × 10.5-mm^2^ rectangular conductor loop. But unlike the circuit depicted in Fig. 5, each parametric resonator in Fig. 6 had a split conductor in the center and a pair of continuous conductors adjacently placed above and below the center conductor. When the conductor gaps in the peripheral edges of the square inductor were filled by Schottky diodes (BAS3005B, Infineon, Germany) as variable capacitors, the resonator had a dipole mode resonance at 301 MHz and a lower frequency butterfly mode resonance at 272 MHz. By filling the gap of the center conductor with a 27-pF chip capacitor, the higher frequency butterfly mode was created at 388.3 MHz. To improve the circuit’s coupling efficiency with the wireless pumping field, the parametric resonator was overlaid on top of a passive resonator with a dimension of 11.5 × 7.1-mm^2^ whose conductor gap was filled by a 3-pF chip capacitor. The thickness of the sandwiching substrate between these two resonators was experimentally adjusted to about 0.6 mm until the dipole mode resonance of the parametric resonator was adjusted to 300.3 MHz, i.e. the Larmor frequency at 7T. Meanwhile, the passive coupler showed up a resonance peak at 689 MHz, which was close enough to the optimal pumping frequency at 688.6 MHz that was the sum of 300.3 MHz and 388.3 MHz.

Similarly, the other resonator in this second row had a 12-pF chip capacitor filling the gap in its center conductor, leading to a butterfly mode frequency at 539.7 MHz. To improve the resonator’s coupling efficiency with the pumping field, the parametric resonator was overlaid on top of a passive resonator with a dimension of 11.5 × 7.1 mm^2^ whose conductor gap was filled by a 2.0-pF chip capacitor. After experimental adjustment of the substrate thickness to about 0.6 mm, the dipole mode resonance was 300.3 MHz for the parametric resonator, while the resonance frequency of the passive coupler was 840.0 MHz that was close enough to the sum of 300.3 MHz and 539.7 MHz.

Subsequently, these two almost identical resonators were partially overlapped and inductively decoupled to create an unsplit resonance peak around 300.3 MHz. The lower butterfly frequency was down shifted to 269.1 MHz due to residual coupling between their butterfly modes. As a result, simultaneous activation of these two resonators required optimal pumping frequency at 569.4 MHz, which was the sum of 300.3 MHz and 269.1 MHz. Again, to improve the pumping power efficiency for simultaneous activation, a rectangular resonator was optionally overlaid on top of the parametric resonators via a 2-mm polyimide substrate. This conductor loop had a dimension of 13.6 × 7.1-mm^2^ and a 3.3-pF chip capacitor filling its conductor gap. As a result, less than 10 mW of pumping power was required on the antenna that was 3-cm separated from the resonators’ circuit edge.

### D. MR IMAGING

**FIGURE 7.**
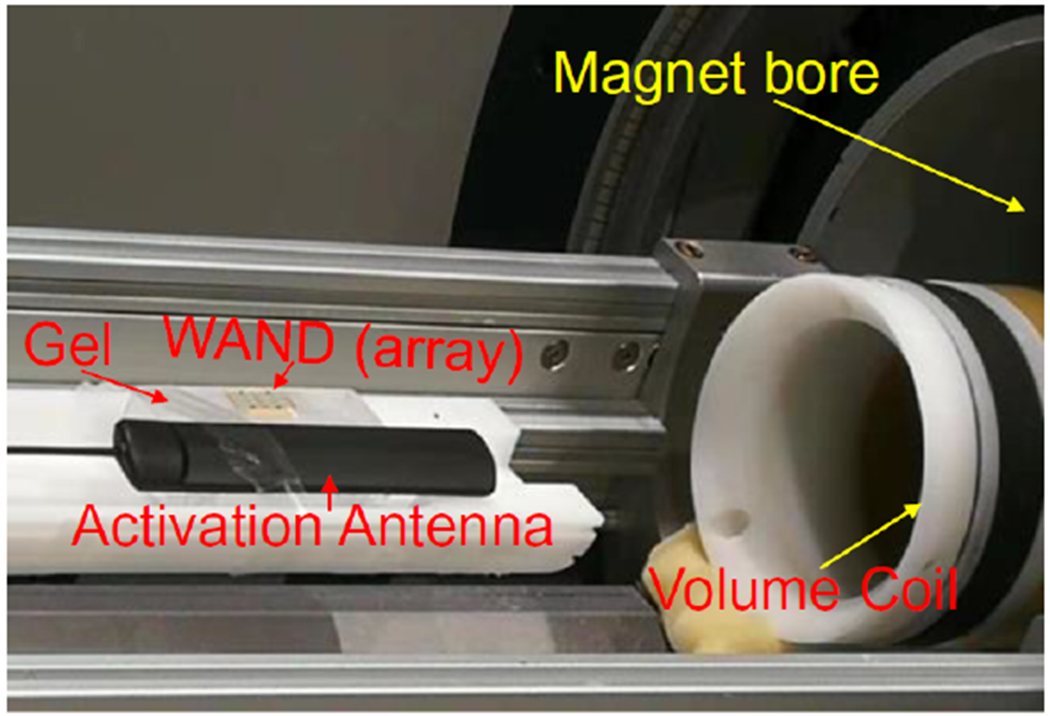
The picture of a WAND (array) placed on top of an agarose gel phantom and activated by an antenna. The volume coil inside the center of magnet would provide RF excitation pulse and receive amplified signals from the WAND.

To evaluate their capability for MR signal amplification, the wireless detectors (or detector array) were placed on the surface of a gel phantom containing 1% agarose. An antenna was placed about 3 cm away from the edge of coupled resonators before the entire assembly was inserted into the inner bore of a 70-mm diameter volume coil that was placed in the center of a 7T magnet (Fig. 7). During RF excitation, the volume coil provided a strong *B*_1_ field to induce large circulating currents that would strongly modulate at least one diode on the parametric resonator. As a result, the circuit’s dipole mode was transiently decoupled from the Larmor frequency, leading to minimum interaction with the B_1_ excitation field. During MR signal acquisition, the pumping signal was turned on to activate the resonator, enabling locally amplified MR signals to be detected by the external volume coil. Both perpendicular and horizontal image slices were acquired using Gradient Refocused Echo sequence, based on the following acquisition parameters TR/TE = 20/10 ms, 15° flip angle, 4.5 × 4.5 cm^2^ FOV, 1 mm slice thickness, 256 × 256 matrix, 25 kHz imaging bandwidth. To selectively activate individual resonators in the first row, the pink-labelled detector in Fig. 5 was activated by a pumping signal at 798.6 MHz applied through a Surface Acoustic Wave filter (STA0415, Sawtron, CA), while the cyan-labelled detector in Fig. 5 was activated by a pumping signal at 917.3 MHz applied through a SAW filter (STA1067A, Sawtron, CA). To simultaneously activate both detectors in Fig. 5, they were activated by a pumping signal at 523.8 MHz applied through a SAW filter (STA1353A, Sawtron, CA). Similarly, individual detectors in the second row (Fig. 6) could be selectively activated by pumping signals at 688.6 MHz and 840.0 MHz applied through surface acoustic wave filters SCT680HT1 and STA0183A respectively. They could also be simultaneously activated at 569.4 MHz through a surface acoustic wave filter (STB0759A, Sawtron, CA).

In all cases, the pumping frequency and pumping power were fine adjusted until an oscillation peak appeared in the center of acquisition window. Subsequently, the pumping power was reduced by 1-dBm below the oscillation threshold to enable 14-dB gain over at least 1-MHz bandwidth (the maximum bandwidth provided by the Bruker MR scanner). To evaluate image sensitivity, two identical images were acquired using the same acquisition parameters and subtracted from each other to obtain the difference image. The normalized intensity of individual pixels was obtained by dividing the average intensity of individual pixels with the standard deviation of the difference image:

(1) $$NI=\frac{\left(\left|S_{1}\right|+\left|S_{2}\right|\right)/2}{std\left(\left|S_{1}\right|-\left|S_{2}\right|\right)}$$

## IV. RESULTS

**FIGURE 8.**
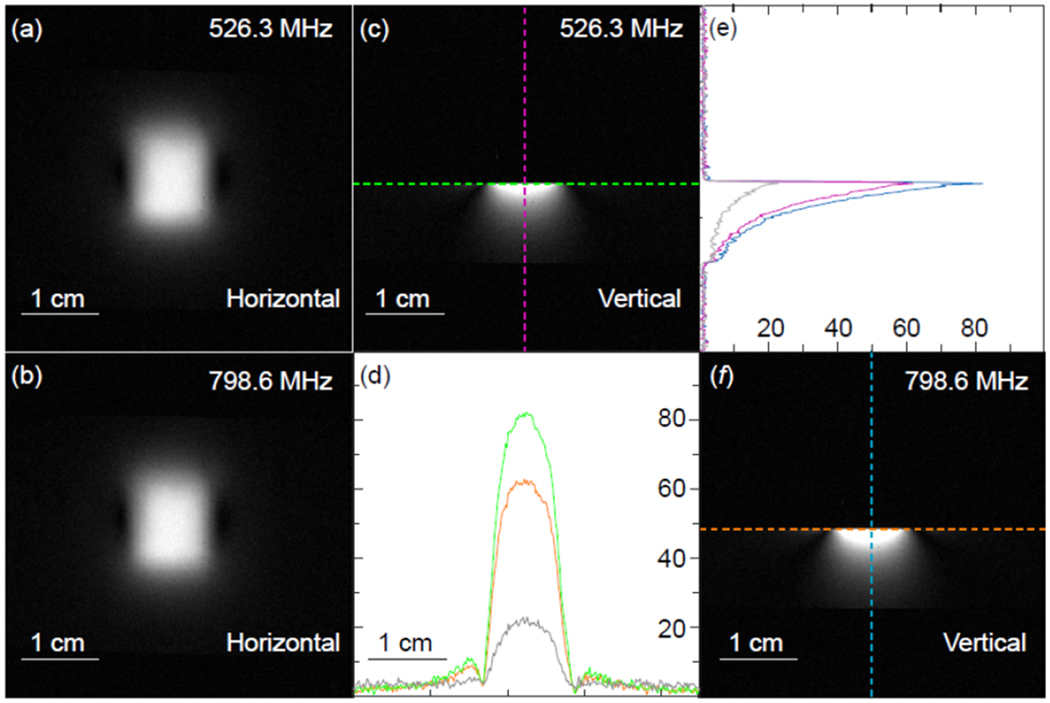
The horizontal and vertical images obtained by a stand-alone resonator that was activated by (a, c) 526.3-MHz pumping signal and by (b, f) 798.6-MHz pumping signal. In (d), the normalized intensity profiles for both vertical images were plotted along the green (for 526.3 MHz) and orange (for 798.6 MHz) lines passing through the edge of the phantom surface. In (e), the normalized intensity profiles for both sagittal images were plotted along the pink (for 526.3 MHz) and blue (for 798.6 MHz) lines that were perpendicular to the phantom surface.

To demonstrate wireless activation of an individual detector by multiple pumping frequencies, each detector was first evaluated in its stand-alone configuration. For example, as shown by the horizontal and vertical images in Fig. 8, similar image profiles were obtained in both orientations when the resonator was activated by pumping signals at 526.3 MHz (Figs. 8a, 8c) and 798.6 MHz (Figs. 8b, 8f). To quantitatively evaluate their relative sensitivity, normalized intensity profiles were plotted along and perpendicular to the surface of the phantom. As shown by the orange curve in Fig. 8d, when the resonator was activated by a pumping signal at 526.3 MHz, it had about 3-fold sensitivity gain over passive coupling (gray line at bottom). Meanwhile, the green curve in Fig. 8d showed about 4-fold sensitivity gain brought by the 798.6-MHz pumping signal over passive coupling. Compared to a surface coil of the same dimension but with direct cable connection to a conventional preamplifier, the WAND could maintain about 80% sensitivity under 798.6-MHz activation and about 64% sensitivity under 526.3-MHz activation. This frequency dependent sensitivity is because according to the Manley Rowe relation [23], the noise factor (*F* = 1 + *ω*_1_/*ω*_2_) was 1.6 when *ω*_1_/*ω*_2_ = 300.3/498.3 under 798.6-MHz activation, while the noise factor was 2.3 when *ω*_1_/*ω*_2_ = 300.3/226 under 526.3-MHz activation. Since the square root of noise factor was inversely proportional to the detection sensitivity, higher pumping frequency would lead to higher detection sensitivity. Similar frequency dependence of the detector sensitivity was also observed in the normalized intensity profiles shown in Fig. 8e.

**FIGURE 9.**
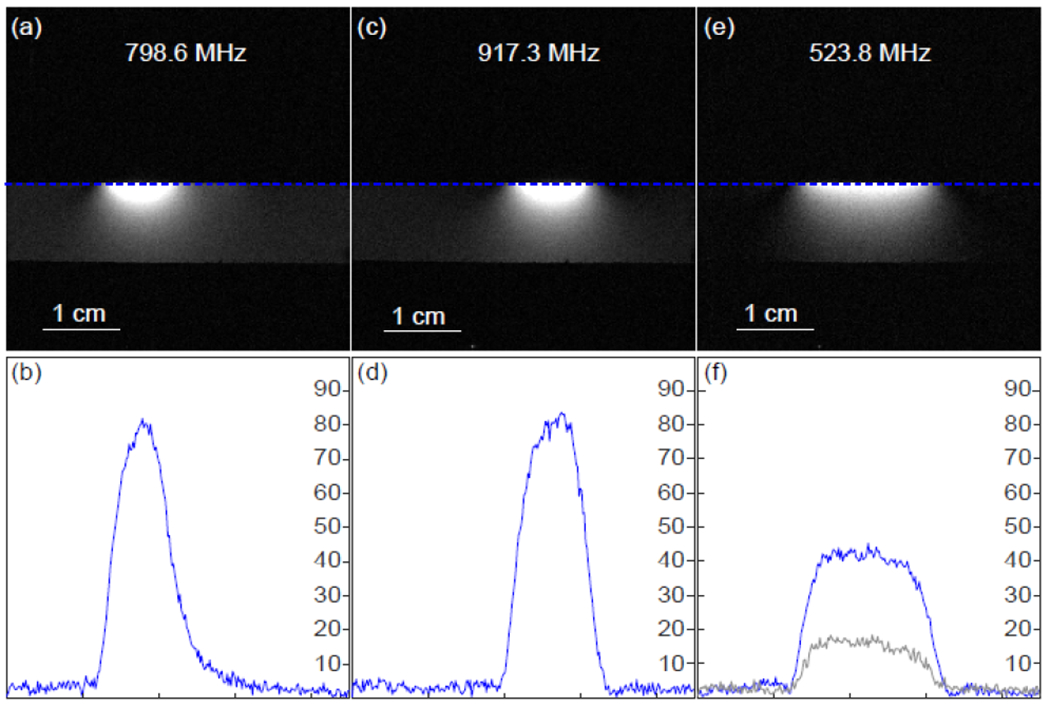
The MR images and the normalized intensity profiles plotted along the edge of the phantom surface, when the concatenated resonators were excited by a pumping signal at (a, b) 798.6 MHz and (c, d) 917.3 MHz for selective activation, or by a pumping signal at €523.8 MHz for simultaneous activation. In (f), the gray curve corresponded to the normalized intensity profile of passive resonators.

When two resonators were concatenated for enlarged FOV, their detection performance was evaluated under selective and simultaneous activation. As shown in Figs. 9a and 9c, resonators on each side of the phantom could be individually activated by pumping signals at 798.6 MHz and 917.3 MHz respectively. By comparing their normalized intensity profiles in Figs. 9b and 9d, both resonators had similar detection sensitivity, due to their similar noise factor as predicted by Manley Rowe relation. Compared to standalone detectors (shown in Fig. 8), individual resonators in the concatenated array showed uncompromised performance. On the other hand, when a pumping signal was applied at 523.8 MHz, both resonators could be simultaneously activated to symmetrically highlight the sample region from both sides. Although less sensitive than individual activation, simultaneous activation was still 3-fold more sensitive than passive coupling (Fig. 9f), and the concatenated resonator pair could maintain about 64% the sensitivity of a cable-connected surface coil of equivalent dimension.

**FIGURE 10.**
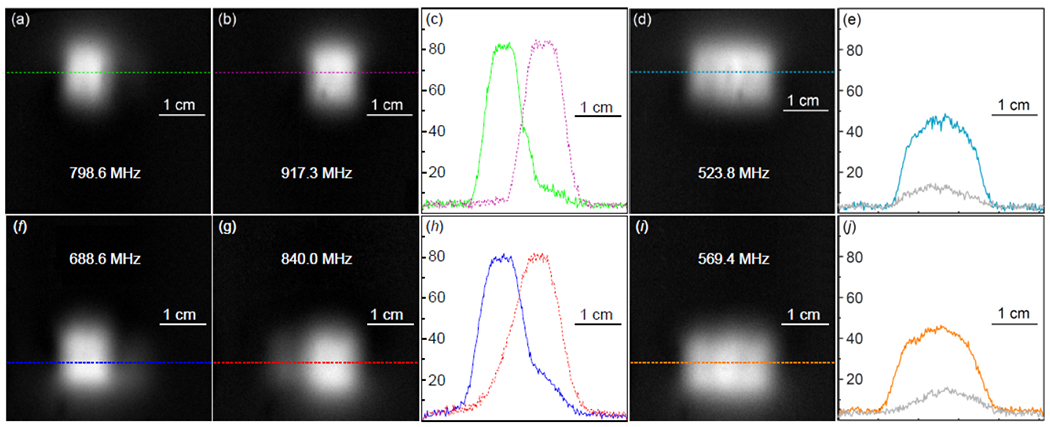
The horizontal images acquired when resonators in the first row were selectively activated by (*a*) 798.6 MHz and (*b*) 917.3 MHz, leading to the normalized intensity profiles (*c*). When both resonators were simultaneously activated by (d) 523.8 MHz, the normalized intensity profile shown as the cyan curve in (e) still had 3-fold sensitivity gain over passive coupling (gray). Similar comparison was also made for resonators in the second row that were (f, g) selectively and (*i*) simultaneously activated, leading to the normalized intensity profiles in (*h*) and (*j*).

When four detectors were arranged into a 2 × 2 element array, both rows could be wirelessly reconfigured between selective and simultaneous activation. As shown in Figs. 10a and 10b, resonators in the first row could be individually activated at 798.6 MHz (Fig. 10a) and 917.3 MHz (Fig. 10b) to individually highlight their detection regions. Similarly, resonators in the second row could be individually activated at 688.6 MHz (Fig. 10f) and 840.0 MHz (Fig. 10g) to individually highlight their detection regions. According to the normalized intensity profiles in Figs. 10c and 10h, all resonators have similar sensitivity within their effective regions. To consecutively highlight the upper and lower portions of the phantom, resonators in the first row were activated by a 523.8-MHz pumping signal (Fig. 10d), before resonators in the second row were activated by a 569.4-MHz pumping signal (Fig. 10i). According to Figs. 10e and 10j, both rows of resonators had symmetric intensity profiles when their constituting elements were simultaneously activated, achieving 3-fold sensitivity gain over passive coupling (gray curves). Because consecutive activation of one row of resonators suppresses residual signal contribution from the other row, the 2 × 2 element array had uncompromised performance compared to the 2 × 1 element array as mentioned above.

## V. DISCUSSION

Wireless Amplified NMR Detectors (WAND) can amplify locally detected MR signals and wirelessly transmit them to remotely coupled external receiver coils. Such a wireless signal link is particularly useful to improve MR detection sensitivity of deep-lying tissues, such as the brain cortex buried beneath a headpost where proximal detection by a cable-connected surface coil is inconvenient to implement. Because the WAND can efficiently amplify MR signals *in situ,* the external receiver coil does not need to be optimized for focal detection. In fact, the same volume coil can be used for both RF excitation and wireless signal reception, without the extra need for a dedicated surface coil. Such a compact arrangement would save precious space inside the narrow bore of a preclinical scanner, enabling future adaption of fiber optics and physiological recording devices for multi-modal imaging. To enlarge the field-of-view beyond the effective range of an individual detector, previous work attempted to concatenate multiple detectors that could be individually activated by unique pumping frequencies. But in order to activate multiple resonators together, multiple pumping signals were applied simultaneously, each of which required individual adjustment of its power and frequency, making the whole adjustment procedure tedious to implement.

In this work, we have developed a wireless reconfigurable detector array, so that only one pumping signal is required to selectively activate individual detectors or simultaneously activate multiple detectors. This is accomplished by making each detector activatable by two pumping frequencies. More specifically, each detector has a dipole mode resonance at *ω_d_*, a lower-frequency butterfly mode resonance at *ω_L_* and a higher-frequency butterfly mode resonance at *ω_H_*. When multiple detectors are concatenated in an 1D array, they share the same *ω_L_* but different *ω_H_*. As a result, when the pumping frequency is adjusted to *ω_d_* + *ω_H_* for selective activation of an individual resonator, parametric amplification is enabled by frequency mixing between the dipole mode and the higher-frequency butterfly mode. Alternatively, when the pumping frequency is adjusted to *ω_d_* + *ω_L_* for simultaneous activation of multiple detectors, parametric amplification is enabled by frequency mixing between the dipole mode and the lower-frequency butterfly mode. Compared to passive coupling, selectively activated resonators can have up to 4-fold sensitivity gain to maintain about 80% detection sensitivity of a cable-connected surface coil (of the same dimension as an individual WAND), thus approaching the sensitivity of the idealized reference standard without wired connections. Although simultaneously activated resonators are somewhat less sensitive than individually activated resonators, they still maintain 3-fold sensitivity advantage over passive coupling. Such ability to switch between simultaneous activation and selective activation will be very useful for interleaved high-resolution single-vessel fMRI inside localized brain cortex [2], [3], [24] and for functional connectivity mapping of global regions [25], [26].

To improve energy efficiency of the higher frequency pumping signal, the parametric resonator is overlaid with a passive resonator that is approximately tuned to the sum frequency of the dipole mode and the “higher butterfly” mode, leading to local enhancement of the pumping field. As a result, the resonator can be activated by less than 10 mW of pumping power applied on an antenna separated from the resonator’s edge by about 3-fold the resonator’s own dimension. Compared to the higher-frequency pumping signal, the lower-frequency pumping signal couples more effectively to the parametric resonator due to its smaller frequency separation from the dipole mode resonance. But in order to further improve energy efficiency of the lower frequency pumping signal, another passive resonator can be optionally introduced that is approximately tuned to the sum frequency of the dipole mode and the “lower butterfly” mode, thus reducing the required level of pumping power from about 50 mW to about 10 mW.

Another favorable feature of the concatenated resonators is their ability to obtain symmetric detection profile when both resonators are activated simultaneously by the same pumping signal. This is because partial overlap of adjacent resonators will decouple their dipole modes but maintaining residual coupling between their butterfly modes. Residual coupling of butterfly modes would not affect electromotive force induced by precessing nuclei spins but would facilitate energy exchange between detectors during parametric frequency mixing process, thus equalizing their gain levels. Of course, simultaneous activation of two resonators will lead to addition of two noise sources. Although less sensitive than an individually activated resonator, the concatenated resonators could still maintain about 64% sensitivity of a cable-connected surface coil (of the same dimension as the concatenated resonators), thus demonstrating their sensitivity advantage for future applications where fMRI signal dynamics needs to be compared between two brain hemispheres [27].

To extend the 1D array into a 2D array, multiple rows of resonators can be parallelly aligned, where resonators in each row have a slightly different circuit pattern to obtain a uniquely identifiable “lower butterfly” frequency. For a 2 × 2 array example, resonators in the first row have a continuous conductor in the center, while resonators in the second row have two continuous conductors that are symmetrically displaced from the center, leading to increased “lower butterfly” frequency compared to the first row. To introduce more rows into the 2D array, it is necessary to fabricate resonators of different lower butterfly frequencies, by geometrically adjusting the distance separation between continuous conductors. On the other hand, it is more convenient to incorporate more detector elements in each row, because the higher butterfly frequency can be fine-tuned by replacing the bridging capacitors without redesigning the circuit pattern. More work is going on to extend the array size in both dimensions, based on the semi-quantitative guidelines provided in the Appendix.

In summary, we have created a wireless reconfigurable amplifier array to enhance MR detection sensitivity of deep-lying regions that are not easily accessible by cable connected surface coils, such as the enclosed cavity buried beneath the headpost. Unlike passive inductive arrays [28], [29] whose individual detector elements cannot be wirelessly controlled, our reconfigurable array can conveniently switch between selective and simultaneous activation to highlight multiple detection regions in real time, enabling convenient multiplexing [30]–[32] of a single-channel MRI scanner without the need for expensive console upgrade. Unlike several other active arrays [33]–[37] that require at least 100 mW of DC power to operate complicated on-board microcontrollers, our reconfigurable array relies on simple nonlinear circuits that can operate by less than 10 mW of wireless power, thus creating a wireless signal link between internal detectors (that are optimized for focal tissues) and the generic detector (that is commonly available on standard MRI scanners). Besides sensitivity enhancement for multi-modal imaging of rodent brains, this wireless reconfigurable detector array will also be useful to improve the operation flexibility of clinical MRI, e.g. when off-the-shelf detectors suitable for adults are too large to sensitively detect focal lesions in children.

## Figures and Tables

**FIGURE 11.**
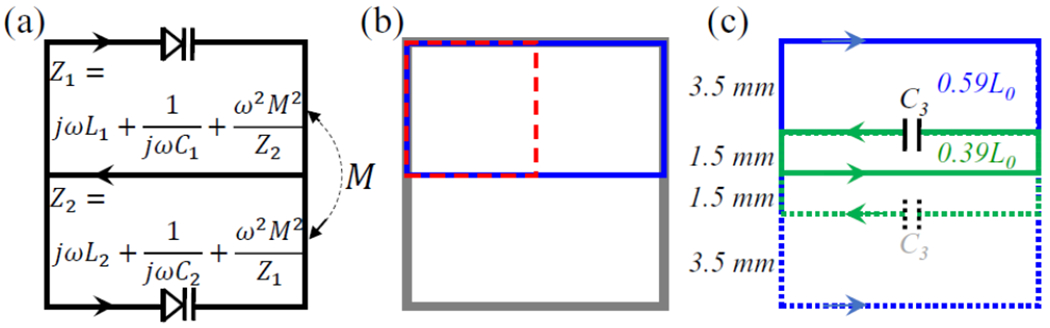
(a) When the square resonator is symmetrically divided by a continuous conductor in its center, the butterfly mode can be analyzed as two half circuits that are inductively coupled together. (b) Compared to the full circuit (gray), the red dashed loop is scaled down by a factor of *S* = 0.5 in both dimensions, leading to scaling down of effective inductance by a factor of 0.5. The blue solid loop is scaled along only one dimension, leading to scaling of effective inductance by a factor of $\sqrt{0.5}$. (c) When two chip capacitors *C*_3_ are bridging the conductor gaps above and below the center, the higher butterfly mode can be approximated as parallelization of the blue solid circuit mesh with effective inductance of 0.59*L*_0_ and the green solid circuit mesh with effective inductance of 0.39*L*_0_, both of which have their current passing through the chip capacitor *C*_3_.

**FIGURE 12.**
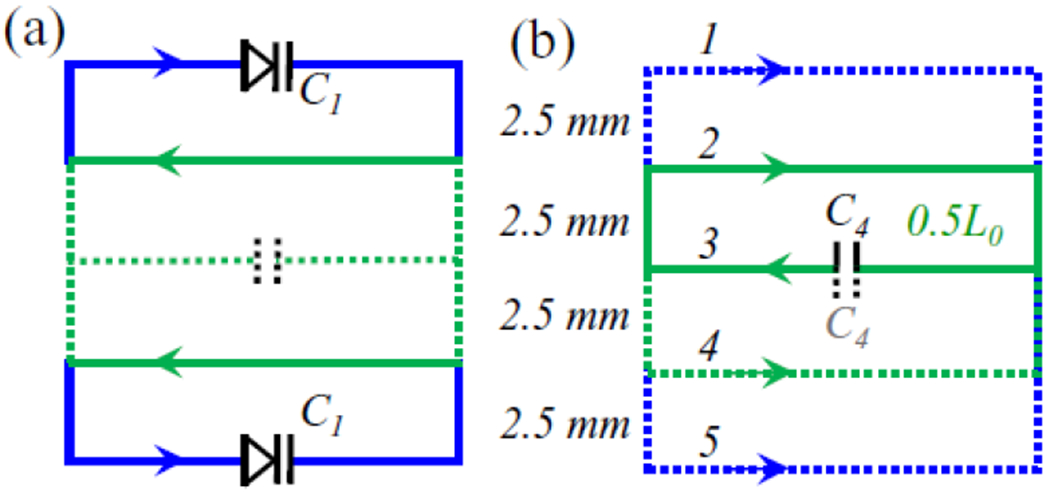
(a) When the continuous conductors (green) are symmetrically displaced from the center by 2.5 mm, the effective height of each half circuit is scaled down by a factor of 0.5, and the effective inductance of each circuit mesh is scaled down by a factor of $\sqrt{0.5}$, making the lower butterfly frequency scaled up by a factor of $1∕\sqrt[{4}]{0.5}$. (b) The higher butterfly frequency is primarily determined by the self-resonance frequency of the green solid loop because the opposite current directions in this loop will cancel its effective interaction with the remaining part of circuit.

## APPENDIX

When a rectangular loop with inductance *L*_0_ is split by two serial capacitors *C*_1_ = *C*_2_ = *C_v_*, the dipole mode resonance frequency is

(A1) $$\omega_{1}=\sqrt{2/\left(L_{0}C_{v}\right)}$$

The loop inductance can be expressed as [38]:

(A2) $$L_{0}=\frac{\mu_{0}}{\pi }\left[\begin{array}{c} a\left(\ln\;\left(\frac{2b}{W\left(1+\sqrt{a^{2}+b^{2}/a}\right)}\right)-0.5\right)+2\sqrt{a^{2}+b^{2}}\\ +b\left(\ln\;\left(\frac{2a}{W\left(1+\sqrt{a^{2}+b^{2}/a}\right)}\right)-0.5\right)+0.447W \end{array}\right]$$

where *a* and *b* are the rectangle’s side lengths, *W* is the width of conductor strip. For a 10 × 10-mm^2^ loop with 0.6-mm strip width, the effective inductance is 28.4 nH. To tune the square resonator to about 300 MHz, *C_v_* ≈ 20 pF is required for each serial capacitor, corresponding to the junction capacitance of zero biased diode (BAS3005B, Infineon).

The butterfly mode is created by connecting the virtual voltage grounds of the dipole mode with a continuous conductor in the center (Fig. 11a). As a result, the entire circuit can be considered as two identical halves concatenated together. Neglecting circuit loss, the effective impedance of the upper circuit half is

(A3) $$Z_{1}\approx j\omega L_{1}+\frac{1}{j\omega C_{1}}+\frac{\omega^{2}M^{2}}{j\omega L_{2}+1/\left(j\omega C_{2}\right)}$$

where *L*_1_ and *L*_2_ are the effective inductance of each half circuit, *M* is their mutual inductance. Resonance occurs when Eq. (A3) is zero, i.e.

(A4) $$0=j\omega L_{1}-\frac{j\omega_{01}^{2}L_{1}}{\omega }+\frac{\omega^{2}M^{2}}{j\omega L_{2}-j\omega_{02}^{2}L_{2}/\omega }$$

where *ω*_01_ and *ω*_02_ are the resonance frequencies of individual half circuits if they were inductively decoupled from each other. Eq. (A4) can be rearranged as:

(A5) $$\frac{L_{1}L_{2}}{\omega^{4}}-\frac{L_{1}L_{2}}{\omega^{2}}\left(\frac{1}{\omega_{01}^{2}}+\frac{1}{\omega_{02}^{2}}\right)+\frac{L_{1}L_{2}-M^{2}}{\omega_{01}^{2}\omega_{02}^{2}}=0$$

According to Vedic theorem, Eq. (A5) should have two solutions for 1/*ω*^2^ that correspond to the inverse square of the dipole resonance frequency $1∕\omega_{1}^{2}$ and the inverse square of the butterfly resonance frequency $1∕\omega_{2L}^{2}$.

(A6) $$\frac{1}{\omega_{1}^{2}}+\frac{1}{\omega_{2L}^{2}}=\frac{1}{\omega_{01}^{2}}+\frac{1}{\omega_{02}^{2}}$$

Or equivalently,

(A7) $$\omega_{2L}=1/\sqrt{1/\omega_{01}^{2}+1/\omega_{02}^{2}-1/\omega_{1}^{2}}$$

For identical circuit halves, *L*_1_ = *L*_2_, leading to

(A8) $$\omega_{01}=\omega_{02}=\sqrt{1/\left(L_{1}C_{v}\right)}$$

Because the dipole mode resonance frequency *ω*_1_ is adjusted to the Larmor frequency for MR signal reception, the butterfly mode resonance frequency *ω*_2*L*_ can be expressed as a function of *ω*_1_ if the relation between *ω*_01_ and *ω*_1_ is known. According to Eq. (A2), when a square inductor is scaled by a factor of S along both dimensions, the loop inductance is also scaled by a factor of *S*. Because scaling along two dimensions is equivalent to consecutive scaling along one dimension, the inductance should be scaled by a factor of $\sqrt{S}$ when the dimension is scaled by a factor of S along one dimension.

(A9) $$L_{1}=L_{0}\sqrt{S}$$

As the example shown in Fig. 11b, when *S* = 0.5, each half circuit will have an inductance of *L*_1_ = *L*_2_ = 0.71*L*_0_, making $\omega_{01}=\omega_{02}=1∕\sqrt{0.71L_{0}C_{v}}$ based on Eq. (A8). As a result, *ω*_2*L*_ can be estimated from Eq. (A7):

(A10) $$\omega_{2L}\approx 1.04/\sqrt{L_{0}C_{v}}\approx 0.74\omega_{1}=222MHz$$

This estimated value of lower butterfly frequency is close enough to the measured value mentioned in Fig. 5.

When the split conductors above and below the center are bridged by two chip capacitors *C*_3_(Fig. 11c), the higher-frequency butterfly mode is created. In this mode, the current circulation in adjacent circuit meshes are opposite. Because the higher butterfly frequency is normally much larger than the self-resonance frequency of the uttermost (blue) circuit branch, the effective impedance of this circuit branch can be approximated as a continuous conductor. Therefore, the higher butterfly frequency is approximately proportional to:

(A11) $$\omega_{2H}\;\propto \;1/\sqrt{C_{3}}$$

Eq. (A11) provides a simple scaling relation for frequency adjustment. To estimate the numerical value of *ω*_2*H*_ for a given C_3_, we need to calculate the effective inductance for the 3.5 × 10-mm^2^ and 1.5 × 10-mm^2^ loops placed in parallel. According to Eq. (A9), the 3.5 × 10-mm^2^ loop has inductance of $\sqrt{0.35}L_{0}=0.59L_{0}$, while the 1.5 × 10-mm^2^ loop has inductance of $\sqrt{0.15}L_{0}=0.39L_{0}$. Parallelization of these two loops leads to an effective inductance of 0.234*L*_0_. When the split conductor is filled by *C*_3_ = 15 pF = 0.75*C_v_*

$$\omega_{2H}=\sqrt{1/\left(0.234L_{0}C_{3}\right)}=2.39/\sqrt{L_{0}C_{v}}\;=1.69\omega_{D}=506.4MHz$$

When the split conductor is filled by *C*_3_ = 10pF = 0.5*C_v_*,

$$\omega_{2H}=\sqrt{1/\left(0.234L_{0}C_{3}\right)}=2.92/\sqrt{L_{0}C_{v}}\;=2.07\omega_{D}=620.1MHz$$

These two estimated frequencies are close enough to the measured values in Fig. 5, thus validating the scaling relation as described by Eq. (A11).

For resonators in row #2, the continuous conductor is displaced by 2.5 mm from the center conductor (Fig. 12a), leading to 0.5-fold scaling of the height in each half circuit. As a result, the effective inductance of each 2.5 × 10-mm^2^ circuit mesh is scaled down by a factor of $\sqrt{0.5}$ compared to the 5 × 10-mm^2^ circuit mesh, making the butterfly frequency roughly scaled up by a factor of $1∕\sqrt[{4}]{0.5}$. Resonators in row #2 will have their lower butterfly frequency scaled up from 226 MHz to 263.5 MHz, which is close enough to the measured value mentioned in Fig. 7. In general, when the height of each half circuit is scaled down by a factor of u, the lower butterfly frequency row #2 can be estimated from the lower butterfly frequency of row #1 according to the following relation.

(A12) $$\omega_{2L2}=\omega_{2L}/\sqrt[{4}]{u}$$

To create the higher butterfly frequency for row 2, another chip capacitor is incorporated to fill the center gap of the center conductor. Again, because the higher butterfly frequency is much larger than the self-resonance frequency of the uttermost (blue) circuit branch, the effective impedance of this circuit branch can be approximated as a continuous conductor, making the higher butterfly frequency approximately proportional to:

(A13) $$\omega_{2H2}\;\propto \;1/\sqrt{C_{4}}$$

The numerical value of higher butterfly frequency can be approximately estimated from the self-resonance frequency of a 2.5 × 10-mm^2^ rectangular inductor (the solid green loop in Fig. 12b) placed in serial with *C*_4_. This is because the opposite current direction in inductors 2 and 3 will effectively cancel their interaction with the remaining parts of circuit with unidirectional current directions. When the split capacitor is 27 pF, *C*_4_ = 13.5 pF = 0.675*C_v_*,

$$\omega_{2H2}=\sqrt{1/\left(0.5L_{0}C_{4}\right)}=1.72/\sqrt{L_{0}C_{v}}=1.22\omega_{1}\;=365MHz$$

When the split capacitor is 12 pF, *C*_4_ = 6pF = 0.3*C_v_*,

$$\omega_{2H2}=\sqrt{1/\left(0.5L_{2}C_{2}\right)}=2.58/\sqrt{L_{0}C_{1}}=1.82\omega_{1}\;=548MHz$$

Again, these two estimated frequencies are close enough to the measured values in Fig. 7.

As a summary, once the dipole mode resonance frequency *ω*_1_ is determined by the Larmor frequency of the scanner (e.g. 300 MHz), the lower butterfly frequency of row #1 can be estimated from Eq. (A10), and the lower butterfly frequency of row #2 can be estimated from the scaling relation in Eq. (A12). Because the higher butterfly frequency can be conveniently adjusted by replacing split capacitors, the simplified circuit models in Figs. 11c and 12b will be sufficient to provide initial frequency estimation, before fine adjustment of the higher butterfly frequency can be performed based on the frequency scaling relations described in Eqns. (A11) and (A13).
